# The PagN protein of *Salmonella enterica *serovar Typhimurium is an adhesin and invasin

**DOI:** 10.1186/1471-2180-8-142

**Published:** 2008-09-08

**Authors:** Matthew A Lambert, Stephen GJ Smith

**Affiliations:** 1 Department of Clinical Microbiology, Trinity College Dublin, St James's Hospital, Dublin 8, Ireland; 2 Department of Microbiology, Moyne Institute, Trinity College Dublin, Dublin 2, Ireland; 3 Institute for Molecular Medicine, Trinity Centre, Trinity College Dublin, St James's Hospital, Dublin 8, Ireland

## Abstract

**Background:**

The *pagN *gene of *Salmonella enterica *serovar Typhimurium is a PhoP-regulated gene that is up-regulated during growth within macrophages and *in vivo *in murine models of infection. The PagN protein displays similarity to the Hek and Tia invasins/adhesins of *Escherichia coli*. Thus far no function has been ascribed to the PagN protein.

**Results:**

Here we show that the outer membrane located PagN protein mediates agglutination of red blood cells and that this can be masked by LPS. When expressed in *Escherichia coli *the PagN protein supports adhesion to and invasion of mammalian cells in a manner that is dependent on cytoskeletal rearrangements. *S. enterica *sv Typhimurium *pagN *mutants display a reduction in adhesion to and invasion of epithelial cells. Finally, we demonstrate that over-expression of PagN in a SPI-1 mutant can partially compensate for the lack of a functional invasasome.

**Conclusion:**

PagN is an outer membrane protein that may contribute to the virulence of *S*. Typhimurium. This protein is a haemagglutinin and contributes to the adherence to mammalian cells. In addition, PagN can mediate high-level invasion of CHO-K1 cells. Previously,*pagN *mutants have been shown to be less competitive *in vivo *and thus this may be due to their lessened ability to interact with mammalian cells. Finally PagN can be added to an ever-growing repertoire of factors that contribute to the pathogenesis of *Salmonella*.

## Background

*Salmonella enterica *serovar Typhimurium infects a wide range of animal hosts and typically causes a self-limiting gastroenteritis [1]. Initial symptoms include nausea and vomiting, which are followed by abdominal pain and diarrhoea. *S*. Typhimurium adheres to intestinal epithelial cells using a myriad of fimbriae including Type 1 fimbriae [2]. Outer membrane proteins such as the plasmid-encoded Rck [3-5] and the OmpD [6] protein also mediate attachment to epithelial cells. Deletion of either *rck *or *ompD *results in decreased invasion of intestinal epithelial cells [5,6]. Upon adhesion the subsequent uptake of *Salmonella *into mammalian cells is a complex process that is coordinated by a series of proteins that are encoded within the SPI-1 and SPI-5 pathogenicity islands [1]. The SPI-1 locus encodes a type three secretion system (T3SS), also known as an invasasome, that delivers effector protein including the *Salmonella *outer proteins (Sop) SopE, SopE2, SopB, the secreted protein tyrosine phosphatase SptP, and the *Salmonella *invasion proteins (Sip), SipA and SipC into cells [1]. These effectors have profound effects on the regulation of actin cytoskeletal structure and on mammalian cell membrane plasticity [7]. The net result of injecting these effectors into the host cell is bacterial-promoted endocytosis [7].

The PhoPQ system regulates the transcription of a multitude of virulence genes of *Salmonella *[8]. This regulatory system is composed of the PhoQ membrane-bound sensor kinase and the PhoP response regulator. In response to specific stimuli, PhoQ modifies the phosphorylation state of the cytoplasmic DNA-binding protein PhoP, increasing its affinity for specific DNA targets [8]. Genes that are PhoP-activated are known as *pags *(PhoP-activated genes) whilst those that are repressed are known as *prgs *(PhoP-repressed genes) [8]. Three different cues have been proposed to activate the PhoPQ system inside macrophages: a mild acidic pH, antimicrobial peptides, and low Mg^2+ ^concentration [8]. The *pagN *gene is a PhoP-activated gene [9]. The *pagN *ORF was originally identified in a Tn*phoA *random-mutagenesis screen of the *S*. Typhimurium strain 14082s [9]. In that study bacteria with active *phoA *gene-fusions that displayed decreased fusion-protein activity on acquisition of the *phoP12 *allele (which results in a PhoP null phenotype) were identified. Through the use of *in vivo *expression technology (IVET), Heithoff *et al*. identified over 100 genes that were specifically expressed during infection of BALB/c mice [10]. These authors showed that *pagN *(also termed *iviVI-A *in that study), although not expressed in *S*. Typhimurium strains grown on typical laboratory medium, was required for survival in BALB/c mice under the conditions of the IVET selection. Subsequently, Conner *et al*. noted a decrease in competitiveness for a mutant with deletions in the region of *pagN*/*iviVI-A *when mixed with its parental strain [11]. Expression of the *pagN *gene has been shown to respond to pH and Mg^2+ ^concentration *in vitro *[12]. As with other *pags*, and in a PhoP-dependent manner, *pagN *expression was induced in conditions of acidic pH and low Mg^2+ ^concentration. Furthermore, expression of *pagN *has been demonstrated to be highly up-regulated within cultured RAW264.7 macrophages and Henle-407 enterocytes [12].

Whilst the function of PagN is unknown it does display similarity to known invasins of *Escherichia coli*. PagN is ~54% similar to the Hek and Tia adhesins/invasins of pathogenic *E. coli *[13-15]. Based on this homology it is possible that PagN may be an adhesin and/or invasin. Here we show that PagN, when expressed in *E. coli*, can agglutinate erythrocytes as well as mediate adhesion to and invasion of mammalian cells. Furthermore *S*. Typhimurium mutants defective in *pagN *display reduced adhesion to and invasion of mammalian cells. Finally, multi-copy expression of PagN can compensate for the loss of the SPI-1 encoded T3SS.

## Results

### PagN is a haemagglutinin but not an autoagglutinin

**Figure 1:**
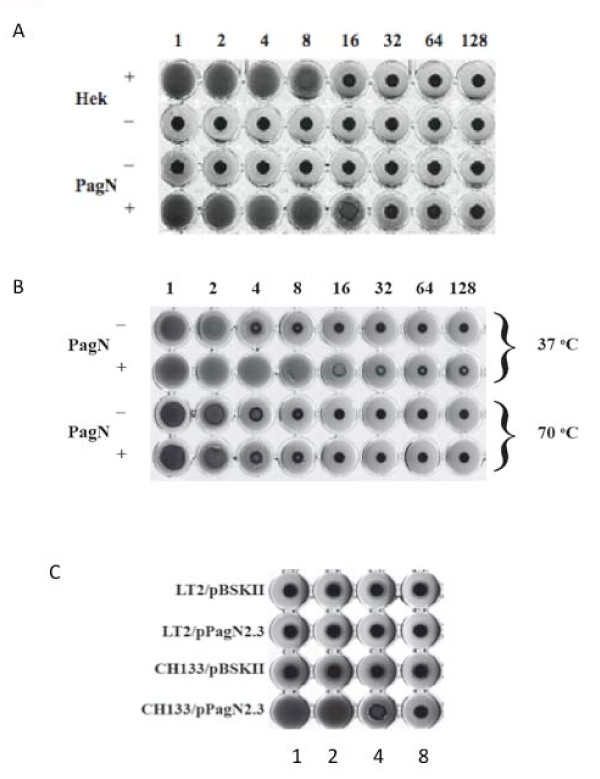
**Haemagglutination by *E. coli *or *Salmonella *expressing PagN**. **(A) **Cultures of *E. coli *strain XL-1 Blue harboring plasmids pML1 (PagN^+^) or pTrc99a (PagN^-^) were induced with IPTG and then mixed with 3% human blood. Overnight cultures of *E. coli *harboring plasmids pHek6 (Hek^+^) and pBSKII+ (Hek^-^) were included as a positive control and vector control respectively. **(B) **Assays were carried out as described in Methods with cultures being incubated at 37°C or 70°C for 30 min prior to the assay. **(C) **The effect of LPS on PagN-promoted agglutination. *S*. Typhimurium strain LT-2 or CH133 harboring either pBSKII+ or pPagN2.3 were grown in MOPS minimal media adjusted to pH 5.8. Haemagglutination assays were performed as described in Methods.

The Hek protein from *E. coli *K1 strain RS218 agglutinates erythrocytes in a heat-resistant manner [13,14]. PagN and Hek are ~54% similar, indicating that PagN may also promote erythrocyte agglutination. To test this hypothesis, a microtitre haemagglutination (HA) assay was performed as described in Methods. *E. coli *K-12 strain XL-1 Blue containing the *pagN *expression-vector pML1 or the empty vector control, pTrc99a, were incubated with human erythrocytes. The expression of PagN conferred upon recombinant *E. coli *the ability to agglutinate erythrocytes (Fig. 1A). Using 3% human blood, a bacterial HA titre of 16 was displayed by PagN-expressing *E. coli*, similar to that of *E. coli *expressing Hek (data not shown). Hek is a heat-resistant agglutinin; heating Hek-expressing cells to 70°C does not reduce agglutination activity [14]. We sought to determine if PagN was also a heat-resistant agglutinin. Unlike Hek, PagN displayed a decrease in HA titre after heating (Fig. 1B). Thus PagN is a heat-sensitive agglutinin.

In addition to promoting haemagglutination Hek is also an autoagglutinin [14]. Autoagglutination can be conveniently measured in a kinetic manner by a spectrophotometric assay. Expression of the Hek protein from pHek6 in *E. coli *XL-1 Blue caused bacteria to settle out of solution at a rate of 0.024 OD_600 nm _units/min whereas bacteria expressing PagN settled at rate of 0.0035 OD_600 nm _units/min almost identical to that of the vector control strain. Thus, *E. coli *expressing PagN did not support autoagglutination.

Next we sought to establish if PagN could support haemagglutination when expressed in multicopy in *S*. Typhimurium. Plasmid pPagN2.3 was introduced into *S*. Typhimurium LT-2 and cultured in minimal MOPS medium. Growth in this medium leads to maximal induction of the PhoP-dependent *pagN *promoter. Expression of PagN was confirmed by SDS-PAGE analysis of outer membrane extracts (data not shown). Surprisingly *S*. Typhimurium/pPagN2.3 did not mediate agglutination of red blood cells (Fig. 1C). Laboratory strains of *E. coli *such as K-12 or B completely lack the O antigen component of lipopolysaccharide (LPS) [16]. It has been reported that porins in *E. coli *are partially obscured by the LPS core and completely blocked by O antigen sugars [17] and indeed in *S*. Typhimurium the O antigen prevents the outer membrane PgtE protease from interacting with its substrate [18]. *S*. Typhimurium has a large O antigen, which may serve to mask PagN thus limiting access to its receptor. To investigate this hypothesis, the plasmid pPagN2.3 was transformed into *S*. Typhimurium strain CH133, a *galE *mutant. The *galE *gene encodes the enzyme UDP-galactose 4-epimerase which is the first enzyme catalyzing O antigen biosynthesis. Therefore *S*. Typhimurium strain CH133 lacks the O antigen component of LPS. Haemagglutination assays using this strain showed that PagN, when over-expressed in a rough strain, was capable of promoting agglutination (Fig. 1C). These data suggest that LPS is capable of 'masking' PagN and preventing functional receptor binding during haemagglutination.

### PagN is an adhesin and invasion

**Figure 2:**
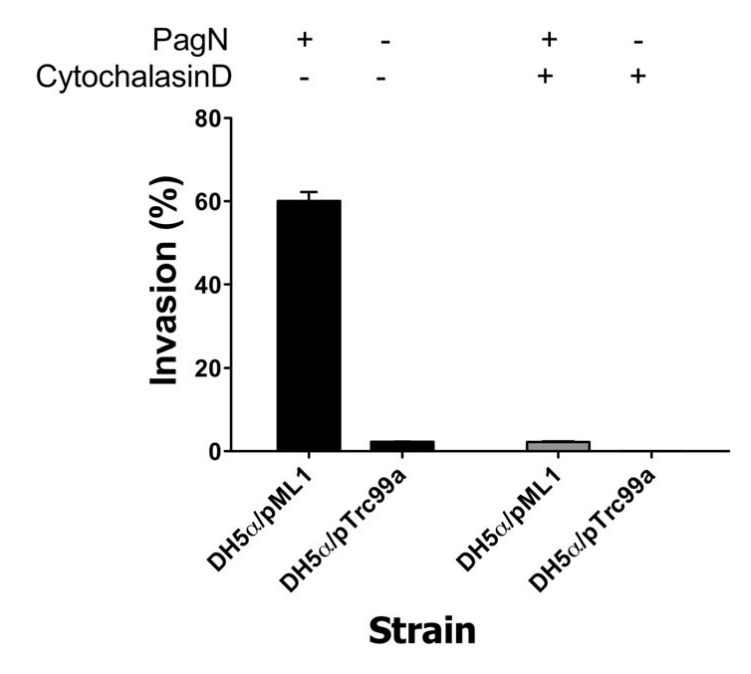
**PagN-promoted invasion of epithelial cells requires actin polymerisation**. *E. coli *DH5α harboring either pTrc99a or pML1 were induced with IPTG and incubated with CHO-K1 cells and invasion levels were measured (black bars). To assess the role of actin filament polymerisation in PagN-mediated invasion, confluent CHO-K1 monolayers were pre-incubated with cytochalasin D (1 μg/ml) for 30 min at 37°C before infection with bacteria (grey bars).

Given that PagN has similarity to both the Tia and Hek invasins we determined if PagN could mediate adhesion to and invasion of epithelial cells. *E. coli *DH5α harboring either pTrc99a or pML1 were induced with IPTG and incubated with CHO-K1 cells. To enumerate the number of invading bacteria, a gentamicin protection assay was performed. Approximately 60% of PagN-expressing bacteria invaded the CHO-K1 cells. Indeed, bacteria that expressed PagN were 27-fold more invasive than bacteria harboring the vector control plasmid (Fig. 2).

Invasion of epithelial cells by bacteria is often accomplished by inducing cytoskeletal rearrangements by manipulating polymerisation of actin. Actin filaments have a fast and slow growing end; cytochalasins inhibit elongation at the fast growing end [19]. Specifically, cytochalasin D affects the polymerisation of actin in two ways: it increases the initial rate but markedly reduces the final extent of the Mg^2+^-induced polymerization process [19]. To assess the role of actin filament polymerization in PagN-mediated invasion, confluent CHO-K1 monolayers were pre-incubated with cytochalasin D (1 μg/ml) for 30 min at 37°C before infection with bacteria. Cytochalasin D was present throughout the invasion assay. Treatment with this inhibitor lead to a 30-fold reduction in invasion, which was comparable to the level seen with the vector control in the absence of cytochalasin D (Fig. 2).

**Figure 3:**
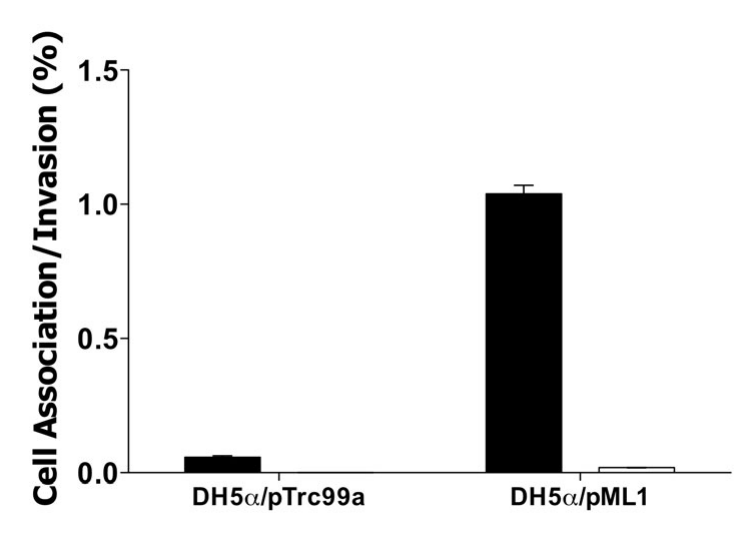
**Interaction of *E. coli *K-12 expressing PagN with HT-29 cells**. *E. coli *DH5α harboring either pTrc99a or pML1 were induced with IPTG and incubated with HT-29 cells and cell association (black bars) and invasion (white bars) levels were measured. Percentage cell association and invasion were calculated as the viable number of bacteria after the assay as compared to the initial input inocula size. The strains tested are indicated. Data represents an average of triplicate wells and standard error bars are shown. Invasion by *E. coli *harboring the vector plasmid was not detectable in this assay.

Whilst CHO-K1 cells provide an accessible and convenient model for studying host-pathogen interactions they do not wholly emulate the human gut; more realistic cell culture models are used to study *Salmonella-*gut interactions, for example HT-29 intestinal epithelial cells [20]. *E. coli *strain DH5α containing the *pagN *expression plasmid pML1 or the vector control pTrc99a were incubated with confluent HT-29 monolayers. Approximately 1% of the bacteria expressing PagN bound to the eukaryotic cells as determined by a cell association assay (Fig. 3). This figure was 6-fold greater than the vector control. Of the bound bacteria that expressed PagN, ~2% had invaded the cell monolayer. No detectable invasion of HT-29 cells by the vector control was observed. Thus, PagN promotes adhesion to HT-29 cells and low level invasion of these cells.

### PagN contributes to *Salmonella *adhesion to and invasion of epithelial cells

**Figure 4:**
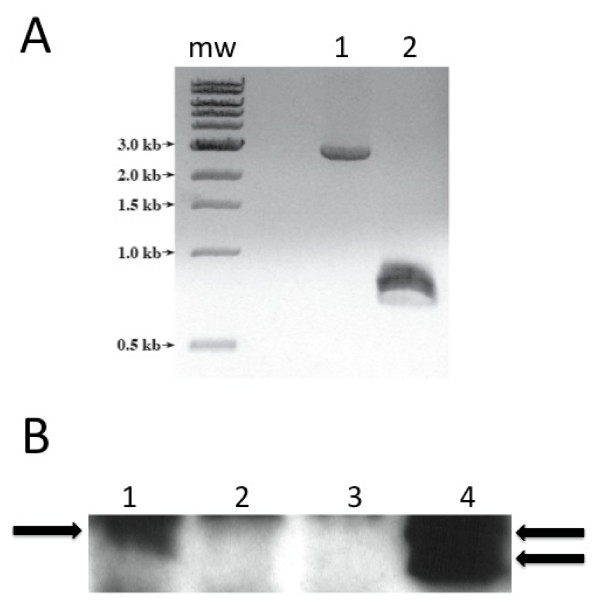
**Characterization of *pagN *mutant strain ML6**. **(A) **Colonies of strains SL1344 and ML6 were boiled and used as a template in a PCR reaction with primers PagNF2 and PagNR2 to amplify the *pagN *ORF. MW indicates molecular size markers. Lane 1 ML6, lane 2 SL1344. **(B) **Sarkosyl-extracted outer membrane proteins were electrophoresed through a 15% gel. Proteins were transferred to PVDF membrane and probed with an anti-PagN antibody. The position of PagN is arrowed. When expressed from a multicopy plasmid PagN was found to migrate as a doublet in common with the Hek protein. Lane 1, SL1344; Lane 2, ML6; Lane 3, ML6 with pBR322; Lane 4, ML6 with pML10.

**Figure 5:**
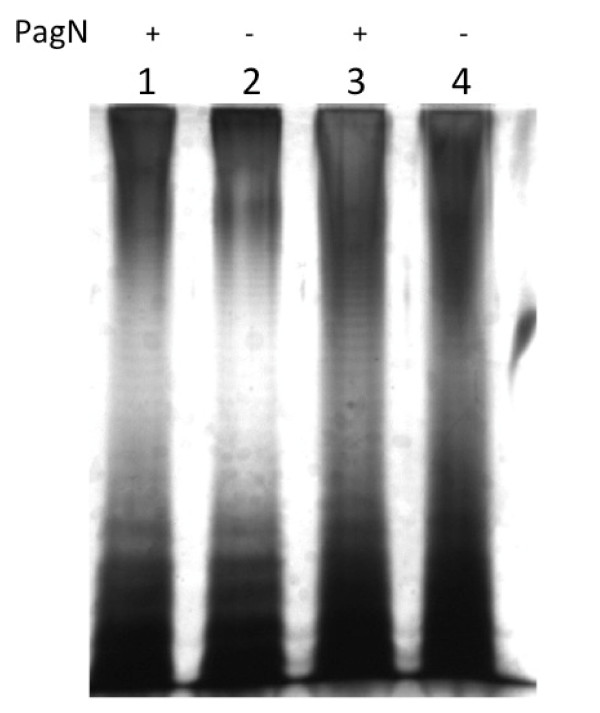
**LPS profiles of *pagN *mutants**. LPS was isolated from *S*. Typhimurium strains as described, electrophoresed through a 12.5% SDS-PAGE gel and the gel was silver-stained. Lane 1, SL1344; Lane 2, ML6; Lane 3, LT-2: Lane 4, MLT-2. PagN status is indicated above the lanes.

A *pagN *mutant strain ML6 (*pagN*^-^) was constructed as described in Methods. This mutant was verified by PCR (Fig. 4A) and the lack of PagN expression in the outer membrane of strain ML6 was verified by immunoblotting (Fig. 4B, lane 2). This mutation could be complemented by expressing PagN from plasmid pML10 (*pagN*^+^) (Fig. 4B, lane 4). The lack of PagN had no effect on the growth of *S*. Typhimurium with both wild-type SL1344 and *pagN *mutant strain ML6 having doubling times of ~45 min in MM 5.8 minimal media. After overnight culture in this medium these bacteria grew to a similar extent having optical densities of ~1.5 OD_600 nm_. Some *pag *gene products such as PagL have been associated with modification of LPS [21]. We thus compared the LPS profiles of SL1344 to ML6 (*pagN*^-^) and LT-2 to MLT-2 (*pagN*^-^) (Fig. 5). In each case there was no difference between the LPS profile of the wild-type and mutant.

The contribution of PagN to the invasion of CHO-K1 cells by *S*. Typhimurium strain SL1344 was determined. Invasion promoted by *S*. Typhimurium strain SL1344 and its *pagN *mutant derivative, ML6, were tested with non-polarized cells in a standard invasion assay. These data established that wild-type *S*. Typhimurium strain SL1344 were internalized in significantly greater numbers than the *pagN *mutant strain ML6. Data from triplicate wells revealed that wild-type levels were reduced from 18.26 ± 2.35% to 9.99 ± 1.37% for a *pagN *mutant (*P *< 0.05). The PagN-defective bacteria displayed a consistent ~2-fold reduction in CHO-K1 cell invasion as compared to the wild-type parent.

**Figure 6:**
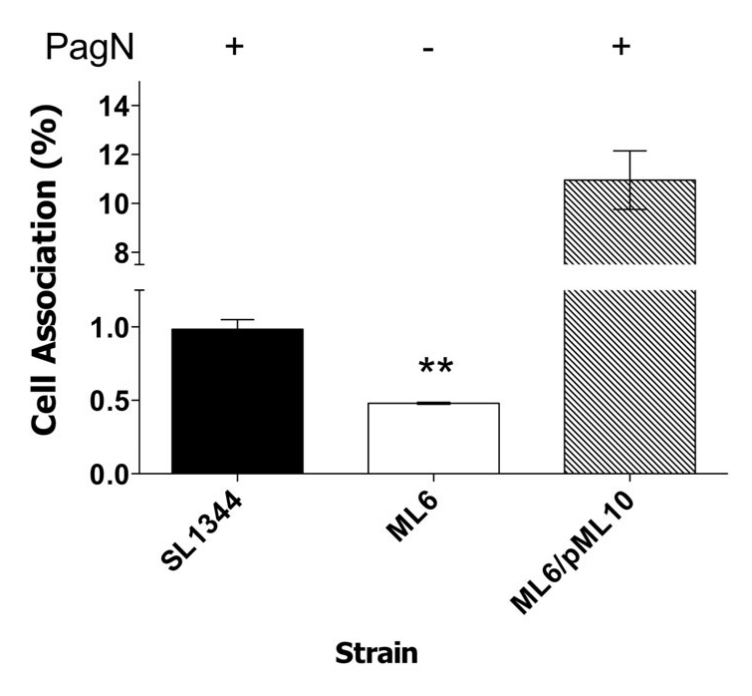
**Adhesion of HT-29 cells by *S*. TyphimuriumSL1344 and a *pagN *mutant**. Cell association levels werecalculated for wild-type *S*. Typhimurium strain SL1344 andthe *pagN *mutant, strain ML6. Levels were also calculated for the *pagN *mutant harbouring the plasmid pML10 (PagN^+^). Data presented are averages of triplicate wells. ** indicates statistical significance, *P *< 0.01.

Adhesion to the HT-29 cell line by *S*. Typhimurium strain SL1344 and adhesion-defects due to the absence of the PagN protein in strain ML6 were investigated. Standard cell association assays were performed comparing the levels of cell association displayed by wild-type and PagN-defective *S*. Typhimurium (Fig. 6). Wild-type bacteria were recovered in higher numbers compared to PagN-defective bacteria (0.98 ± 0.093% as compared to 0.48 ± 0.01%). Complementation of the *pagN *mutation with the *pagN*-containing multi-copy plasmid pML10 increased cell association to 10-fold greater than that exhibited by wild-type *S*. Typhimurium.

**Figure 7:**
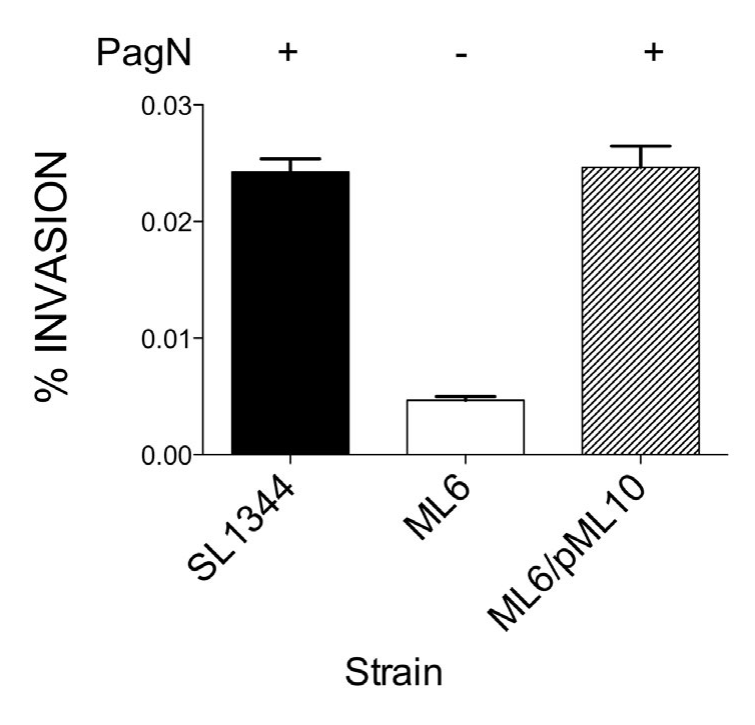
**Invasion of HT-29 cells by *S*. Typhimurium SL1344 and a *pagN *mutant**. Invasion levels were calculated for wild-type *S*. Typhimurium strain SL1344 and *pagN *mutant derivative ML6. Levels were also calculated for strain ML6 harboring pML10 (PagN+) or the vector control pBR322. Data represents an average of triplicate wells.

Furthermore, the *pagN *mutant strain ML6 in comparison to SL1344 displayed a five-fold reduction in invasion of HT-29 cells (Fig. 7). The overall levels of invasion in all cases were low since the bacteria were cultured in MM 5.8 medium. Cultivation in this medium results in maximal expression of PagN but the expression SPI-1 system is greatly reduced [22]. The decrease in invasion due to the loss of PagN could be complemented to wild-type levels with plasmid pML10. The introduction of pML10 into strain ML6 resulted in a 10-fold increase in adhesion over wild-type levels (see above). However, a corresponding 10-fold increase in invasion levels by pML10/ML6 was not observed. This may be due to saturation of the host cell functions that PagN might use to induce invasion. In summation PagN can contribute to adherence and to low level invasion in the two cell lines tested by *S*. Typhimurium SL1344.

**Figure 8:**
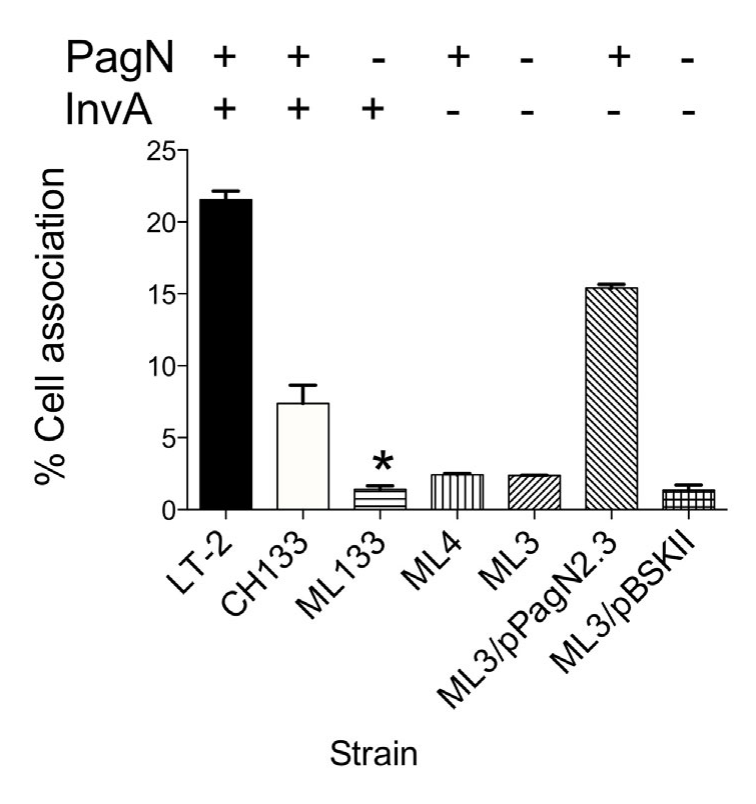
**Invasion of CHO-K1 cells by rough *S*. Typhimurium lacking a functional SPI-1-encoded T3SS**. Invasion levels were calculated for rough *S*. Typhimurium strains with a functional T3SS, CH133 (PagN^+^) and ML133 (PagN^-^) and strains without a functional T3SS, ML4 (PagN^+^, InvA^-^) and ML3 (PagN^-^, InvA^-^). Levels were also calculated for ML3 harboring pPagN2.3 or the vector plasmid pBSKII+. Data represents an average of triplicate wells and standard error bars are shown. * indicates statistical significance of *P *< 0.05.

Haemagglutination by *Salmonella *expressing PagN was only observed in rough strains (see above). This was not the case for adherence to or invasion of mammalian cells since we could observe defects in these phenotypes for *pagN *mutants in the smooth *S*. Typhimurium strain SL1344. Nevertheless we examined if a rough strain might facilitate enhanced invasion and/or adhesion by PagN. In these studies we made use of strain CH133 a rough mutant of *S*. Typhimurium LT-2 (a rough mutant of strain SL1344 was not readily available for these analyses). A *pagN *rough mutant strain, ML133, displayed approximately a five-fold decrease in cell association which was greater than the defect due to loss of *pagN *in smooth strains (Fig. 8). The magnitude of the decrease in cell association in *pagN *rough mutants was similar to that seen for the loss of the SPI-1 encoded T3SS by mutating *invA *in strain ML4. Moreover, there was no additive effect of deleting both *pagN *and *invA*. Intriguingly, loss of both *pagN *and *invA *in strain ML3 could be complemented by introducing the PagN multicopy plasmid pPagN2.3. Indeed, the level of cell association for pPagN2.3/ML3 was greater than that of CH133.

## Discussion

**Figure 9:**
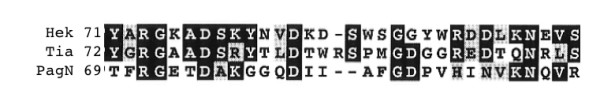
**Comparison of loop2 of Hek with the corresponding sequences in PagN and Tia**. Loop 2 of Hek was aligned with the corresponding sequences in Tia and PagN using ClustalW. The amino acid numbering is with respect to the sequence of the mature protein in each case. Identical residues are shaded black, whilst semi-conserved are shaded grey.

The *pagN *gene is widely distributed throughout the *Salmonella *[23] and confers a competitive advantage *in vivo *[11]. However, the function of PagN is unknown. Deletion of the *Salmonella enterica *subspecies I-specific centisome 7 genomic island (on which *pagN *resides) leads to decreased association with human cells [24]. We have observed that the PagN protein is located in the outer membrane of *S*. Typhimurium. Together with its similarity to the Hek and Tia invasins/adhesins we postulated that it might serve a similar role in *S*. Typhimurium. When ectopically expressed in *E. coli *K-12, PagN could mediate haemagglutination in a manner similar to that observed for the Hek protein. In contrast to Hek, PagN is not a heat-resistant agglutinin and does not promote autoagglutination. This suggests that the folding of PagN is sensitive to heating and that this affects its biological activity. Whilst Hek and PagN are similar proteins it is likely that this similarity is restricted to the conserved membrane spanning regions of the proteins. Hek is predicted to have eight membrane-spanning regions and four exposed extracellular loops [13]. A similar arrangement is likely for PagN (Lambert MA and Smith SGJ, in preparation). The second extracellular loop of Hek is absolutely required for autoagglutination [13]. The corresponding sequence in PagN is markedly different with only nine identical residues out of thirty (Fig. 9). Thus it is likely that PagN does not have the requisite sequence for bacterial cell:cell interactions. Furthermore, loop 2 of Hek is absolutely required for haemagglutination [13]. Obviously the amino acid sequence of loop 2 of Hek renders it resistant to heating whereas the corresponding sequence in PagN is different and this may underpin its heat-sensitivity (Fig. 9). Interestingly, the Tia protein is also highly divergent at this sequence location (Fig. 9).

PagN failed to promote haemagglutination when overexpressed in *S*. Typhimurium LT2. This inability to promote haemagglutination may be explained by the differences between the LPS of *E. coli *laboratory strains and *S*. Typhimurium strain LT2. Laboratory strains of *E. coli *such as K-12 and B completely lack the O antigen component of LPS [25] whereas *S*. Typhimurium has a large O antigen containing OAc, Abe, Man, Gal, and Glc. When over-expressed in the rough *S*. Typhimurium strain CH133, PagN promoted haemagglutination. PagN contains fifteen positively charged amino acids that we predict to be surface-exposed. It is likely that these amino acids might interact with negatively charged LPS thus masking PagN and preventing interactions between the protein and its mammalian target.

Expression of PagN in *E. coli *resulted in adhesion to and invasion of mammalian cell lines. High levels of invasion promoted by PagN were observed for CHO-K1 cells. Invasion of HT-29 cells by *E. coli *expressing PagN was much lower. It may be that the receptor for PagN is less abundant in HT-29 cells, expressed by fewer cells or possibly that the receptor is not as accessible in this cell line. Another possibility may be that the bacterial cells do not survive within the environs of HT-29. HT-29 is known to produce antibacterial peptides and these are deleterious for *E. coli*. HT-29 cells express human beta defensin 1 [26] and also up-regulate expression of LL-37 in response to *E. coli *[27]. A similar difference in the efficiency of invasion of CHO-K1 cells versus the T84 intestinal epithelial cell line was previously noted for the Hek protein [14].

Mutation of *pagN *in *S*. Typhimurium resulted in a decrease in invasion of and adhesion to CHO-K1 and HT-29 cells. Indeed, this defect was more pronounced for rough strains. Though mutation of *pagN *did not completely abrogate adhesion and invasion the magnitude of the effect is of the same order as that seen for OmpD [6] and Rck proteins [5]. Thus outer membrane proteins such as PagN may play an incremental role in *Salmonella *interactions with host cells. The *pagN *gene is activated by PhoP and is maximally expressed intracellularly as observed by gene fusion [11,12] or microarray analysis [28]. Thus bacteria that exit epithelial cells or macrophages might be expected to have an optimal level of expression and this might facilitate subsequent interactions with mammalian cells that the pathogen encounters. The fact that PagN can functionally substitute for the lack of a functional T3SS may have biological relevance. Within macrophages the SPI-1 T3SS is strongly downregulated [28] and *Salmonella *that exit such cells may have to rely on PagN to facilitate subsequent interactions with cells until such time as the T3SS is maximally expressed.

The primary sequence of PagN has similarity to the Tia and Hek adhesions/invasins. Both Tia and Hek bind heparin sulphate proteoglycan (HSPG) [13,29]. Studies are currently in progress to establish if PagN utilizes HSPG for adhesion/invasion.

## Conclusion

We have shown that PagN can mediate interactions with mammalian cells when expressed in *E. coli *and *S*. Typhimurium. *pagN *is upregulated *in vivo *and contributes to the competitiveness of *S*. Typhimurium. This may be due to the adhesive and invasive characteristics of PagN. PagN in multicopy can complement an *invA *mutation. PagN may thus prove to be a useful tool for studying *Salmonella *in the intracellular environment in the absence of the SPI-1 T3SS.

## Methods

### Bacterial strains, plasmids and culture conditions

All *E. coli *and *S*. Typhimurium strains used in this study are described in Table 1. Plasmids are listed in Table 2. *E. coli *were routinely cultured in Luria (L) broth or on L agar. For optimal expression of the *pagN *promoter in *Salmonella *bacteria were grown in MOPS minimal media adjusted to pH 5.8 (MM 5.8). Where appropriate the following antibiotics were added to media: carbenicillin (50 μg/ml), chloramphenicol (10 μg/ml), spectinomycin (100 μg/ml).

**Table 1:** Bacterial strains used in this work

Strain	Relevant Features	Reference
***S*. Typhimurium**		
CH133	LT-2 *galE503*	[34]
LT-2	Wild-type strain	ATCC
MLT-2	LT-2 *pagN*::*spc*	This study
ML133	LT-2 *galE503 pagN*::*spc*	This study
ML3	LT-2 *galE503 invA*::*cat pagN*::*spc*	This study
ML4	LT-2 *galE503 invA*::*cat*	This study
ML6	SL1344 *pagN*::*spc*	This study
SL1344	Wild-type strain	ATCC
		
***E. coli***		
DH5α	F' *endA1 hsdR17 *(*rk*^-^*mk*^+^) *glnV44 thi-1 recA1 gyrA *(Nal^r^) *relA1 *Δ(*lacIZYA-argF*)U169 *deoR *(Φ*80dlacΔ *(*lacZ*)M15)	Invitrogen
XL-1 Blue	*recA1 endA1 gyrA96 thi-1 hsdR17 supE44 relA1 lac *[*F' proAB lac*^*q*^*ZΔM15 *Tn*10 *(Tet^r^)]	Stratagene

**Table 2:** Bacterial plasmids used in this work

Plasmid	Relevant Features	Reference
pACYC184	Cm^r ^Tet^r ^General cloning vector	[35]
pBSKII+	Ap^r ^ColE1 replicon	Stratagene
pBR322	Ap^r ^Cloning vector	[36]
pHek6	Apr *hek *gene in Pbskii+	[14]
pHP45Ω	Ap^r ^Spc^r ^pMB1 replicon	[37]
PInvA	Ap^r ^*invA *gene with 500 bp flanking DNA cloned into pBSKII+	This study
pInvAKO	Ap^r ^*invA*::*cat *gene with 500 bp flanking DNA cloned into pBSKII+	This study
pKOBEGA	Ap^r ^Temperature-sensitive plasmid carrying the *red *and *gam *genes of λ-phage under control P_*araBAD *_promoter	[32]
pMALc2	Ap^r ^MBP fusion vector	New England Biolabs
pML1	Ap^r ^*pagN *ORF inserted into pTrc99a	This study
pML4	Ap^r ^*pagN *ORF flanked by *Nco*I *and Bam*HI *sites *inserted into pBSKII+	This study
pML7	Ap^r ^*pagN *ORF, excluding the DNA corresponding to the signal sequence of the PagN protein, inserted into pMAL-c2	This study
pML10	Ap^r ^*pagN *gene inserted into pBR322	This study
pPagN2.3	Ap^r ^*pagN *gene with 500 bp flanking DNA cloned into pBSKII+	This study
pPagNKO	Ap^r ^Spc^r ^*pagN*::*spc*	This study
pTrc99a	Ap^r ^Cloning vector with an IPTG-inducible P_*trc *_promoter	[38]

### Eukaryotic cell lines and growth conditions

All cell lines used were obtained from ATCC (Manassas, VA, U.S.A.). The mammalian cells lines used were HT-29 (ATCC HTB-38) and CHO-K1 (ATCC CCL-61). CHO-K1 cells were grown in a 1:1 mixture of DMEM and Ham's F12 medium supplemented with 10% (v/v) heat inactivated fetal bovine serum (FBS) (Life Technologies) at 37°C in 5% CO_2_. HT-29 cells were grown in McCoy's medium supplemented with 10% (v/v) FBS.

### Recombinant DNA techniques

Plasmid DNA was isolated using the Genelute Plasmid Miniprep kit (Sigma-Aldrich) or the Qiagen Plasmid Midi kit. Restriction endonucleases were purchased from New England Biolabs and used according to the manufacturer's instructions. Standard methods were used for the ligation of DNA fragments and transformation of plasmid DNA [30,31]. The synthesis of oligonucleotide primers and DNA sequencing was performed by MWG Biotech, Ebersberg.

### Cloning of the *pagN *gene

The *pagN *ORF from *S*. Typhimurium strain LT2 and 500 bp of DNA flanking the ORF were amplified by PCR using primers PagNF (5'-AGA TAA TTG CTC GCC ATT CG-3') and PagNR (5'-ATG GAG GGT TCC AGA TCT CC-3'). The purified *pagN *PCR product was cloned into pBSKII + cloning vector (pBSKII+) cut with *Eco*RV creating the multi-copy plasmid pPagN2.3. The pPagN2.3 plasmid was sequenced to confirm the DNA sequence of the insert (Table 1). The *pagN *gene and putative promoter region was excised from pPagN2.3 using *Psh*AI and *Sac*I and the resulting DNA fragment was ligated into vector pBR322. The structure of the resulting plasmid was confirmed by restriction endonuclease digestion and the recombinant was designated pML10 (Table 1).

The *pagN *ORF was amplified with primers pagNF2 (5'-GCT AGG ATC CCG ATA GTG TTT AAA AGG CG-3') and pagNR2 (5'-GCC TCC ATG GAA AAC TTT GCA GTC TGC-3'). The resultant PCR product was digested with *Bam*HI and ligated into plasmid pBSKII+ digested with *Bam*HI and *Eco*RV. The resulting plasmid was sequenced to confirm the sequence of the insert and the plasmid was designated pML4. The PagN expression vector pML1 was constructed by excising the *pagN *ORF from pML4 using *Nco*I and *Bam*HI. This DNA fragment was cloned into pTrc99a, placing it under the control of the IPTG-inducible P_trc _promoter.

The *pagN *ORF was amplified by PCR using the primer set pagNF2 and PagNR3 (5'-GCT ATC TAG ACG ATA GTG TTT AAA AGG CG-3') and *S*. Typhimurium strain LT2 genomic DNA as a template. The PagNR3 primer had an *Xba*I site incorporated into it, resulting in a unique *Xba*I site at the 3' end of the *pagN *ORF. The PCR product was digested with *Xba*I and *Bsa*BI. The *Bsa*BI site is at nucleotide position 75 of the *pagN *ORF, corresponding to amino acid residue number 26. Plasmid pMALc-2 (New England Biolabs) was digested with *Eco*RI and the 5' overhangs were blunted by digestion with Mung Bean Nuclease and was then digested with *Xba*I. The digested PCR product was cloned downstream, and in frame with the *malE *gene. The recombinant plasmid was sequenced to confirm the DNA sequence of the insert and named pML7. SDS-PAGE analysis of *E. coli *XL-1 Blue containing the pML7 plasmid revealed one major inducible protein with an apparent molecular weight of ~65 kDa, corresponding to the predicted size of the MBP-PagN fusion protein. An anti-PagN serum was produced by inoculation of rabbits with the MBP-PagN protein fusion.

### Cloning of the *invA *gene

A 1499-bp fragment of the 2058-bp *invA *gene of *S*. Typhimurium strain LT2 was amplified by PCR using the primers invA-allele-F (5'-CAA ACG CTG CAA AAC TTC AG-3') and invA-allele-R (5'-TTG ATT TCC TGA TCG CAC TG-3'). The PCR product was ligated into the pBSKII+ plasmid that had been digested with *Eco*RV creating the plasmid pInvA

### Disruption of the *pagN *and *invA *genes of S. Typhimurium

An interrupted *pagN *gene was constructed *in vitro *and transferred to the bacterial chromosomes using an allele-replacement system based on the gene products of the λ phage *red *operon. The λ-phage *redγβα *genes were supplied on the temperature sensitive pKOBEGA plasmid under the control of the arabinose-inducible P_BAD _promoter [32]. A 2.2 kb spectinomycin resistance encoding fragment from the pHP45Ω plasmid was excised using *Hind*III, blunted using the DNA polymerase I large (Klenow) fragment and ligated into a *Bsa*BI site 233 bp from the start of the *pagN *ORF in pPagN2.3 yielding plasmid pPagNKO. The disrupted *pagN *gene from pPagNKO was amplified by PCR and transferred to *S*. Typhimurium chromosome as previously described [32]. The *invA *gene of *S*. Typhimurium strain LT-2 was interrupted in a similar manner. A 2.0 kbp chloramphenicol resistance cassette, excised from pACYC184, was used to interrupt the *invA *gene of plasmid pInvA, resulting in the plasmid pInvAKO. The disrupted *invA *gene from pInvAKO was amplified by PCR and transferred to the *S*. Typhimurium chromosome as described above.

### Haemagglutination assays

The ability of bacterial strains to agglutinate erythrocytes was determined using a 3% (v/v) suspension of human blood group A containing 100 mM mannose. Mannose was added to block adhesion by type 1 fimbriae. Bacterial cultures were harvested by centrifugation at 15,800 × *g *for 1 min and resuspended in PBS to an optical density of 1.0 at 600 nm. The bacteria were then serially 2-fold diluted with PBS in a final volume of 100 μl in a 96-well microtiter plate. An equal volume of the 3% blood suspension was added to each well and the plate was incubated at room temperature for 2 hours or at 4°C overnight to allow un-agglutinated erythrocytes to settle out of suspension. Agglutination was seen as a diffuse carpet of erythrocytes spread over the whole well surface. Non-agglutinated cells were seen as a tight button in the centre of the wells.

### Autoagglutination

Overnight cultures were harvested by centrifugation, resuspended in PBS and normalized to an optical density of 8 OD_600 nm_. Samples were taken from the surface of the cultures at regular intervals to determine the OD_600 nm_. Assays were performed in duplicate and the rate of autoaggregation was determined by the mean decrease in optical density over time. Rates of autoaggregation were determined using Kaleidagraph software.

### Preparation and analysis of whole cell lysates and membrane proteins

Samples enriched for outer-membrane proteins were prepared as previously described [13]. Proteins samples were separated by SDS-PAGE using the method of Laemmli and visualized following staining with coomassie brilliant blue R-250 or were transferred to Immobile-P PVDF membrane (Millipore), processed for western blotting, probed with an anti-MBP-PagN antibody and the blot was developed using the SuperSignal West Pico chemiluminescent HRP substrate (Pierce).

### Preparation and analysis of LPS

Overnight cultures (2 ml) of the bacteria to be examined were grown in the presence of appropriate antibiotics. 1.5 ml of each culture was collected by centrifugation at 19,000 × *g *for 2 min. Bacteria were resuspended in 100 μl of 2× Laemmli buffer [33]. Lysis of resuspended bacteria was achieved by boiling the samples for 10 min at 100°C. Lysed bacteria were cooled on ice for 5 min. All protein present in the sample was digested with 20 μl of Proteinase K (10 mg/ml), leaving the LPS component of the outer membrane intact. Digestion of protein was carried out at 60°C for 1 h. A 1:6 dilution of each sample was prepared in Laemmli buffer. As before samples were stored at -20°C and boiled for 5 min prior to use. LPS samples separated by SDS-PAGE were fixed overnight in fixing solution (25% (v/v) isopropanol, 7% (v/v) acetic acid). Gels were then oxidized for 5 min in oxidation buffer (0.7% (v/v) periodic acid in 2.67% (v/v) fixing solution) and subsequently washed four times for 15 min in dH_2_O. Gels were then stained for 10 min in freshly prepared staining solution 0.8 M NaOH (0.1 M), 2% NH_4_OH (v/v), 3% (w/v) AgNO_3_). Excess stain was washed away with 3 × 10 min washes with dH_2_O. Gels were subsequently placed in developing solution (0.04% (v/v) formalin, 2% (w/v) Na_2_CO_3_). Image development was stopped with 10% (v/v) acetic acid.

### Cell association and invasion assays

Cell association and invasion assays were performed as previously described [15]. CHO-K1 cells were seeded into 12 well trays at densities of 3.0 × 10^4^, while HT-9 cells were seeded at a cell density of 2.5 × 10^5 ^cells/well. Cells were grown to confluence.

*Salmonella *strains used in these assays were grown overnight in a MOPS-based minimal media adjusted to pH 5.8. *E. coli *strain DH5α was grown overnight, and subsequently sub-cultured at a 1:50 dilution in 10 ml. At an OD_600 nm _of 0.4–0.6, PagN expression was induced with IPTG (1 mM). Bacteria were grown for a further 3 h. Prior to their addition to confluent mammalian cells, bacteria were washed with PBS and diluted 1:500 in warm tissue culture medium containing D (+)-mannose (100 mM). Mammalian cell monolayers were washed once with warm PBS and bacteria-containing medium (1 ml) was added to each well. The infected cells were centrifuged at 600 × *g *for 5 min to initiate contact between bacteria and the mammalian cells and to synchronize the time of infection. Cells were incubated at 37°C in 5% CO_2 _for 1 h to allow adhesion to and invasion of the cultured cells. Infected monolayers were washed thrice with warm PBS to remove any non-adherent bacteria. To determine the total number of cell-associated bacteria the monolayer was disrupted by treatment with 0.1% Triton X-100 and the released bacteria were enumerated by spreading serial dilutions on agar plates. To determine the number of intracellular bacteria, a standard gentamicin protection assay was performed. Following the 1 h infection, cells were incubated with medium containing gentamicin (100 μg/ml) for 90 min at 37°C in 5% CO_2_, washed with PBS, disrupted with 0.1% Triton X-100 and the released bacteria enumerated as before. The total viable counts of the bacterial inocula were also determined and the cell-association or invasion efficiencies were expressed as the percentage of bacteria recovered from triplicate wells. The student t-test was performed where appropriate. Experiments were performed at least three times and data from a typical experiment is presented.

## Authors' contributions

MAL performed the majority of the research. SGJS performed some of the research. MAL and SGJS analyzed results and wrote the paper. SGJS conceived the study, supervised the research and secured funding for this research.
